# Fioreze C, Dias RG, Dóri LV, Bregolin L, Comin MD. Sintomas de ansiedade e depressão em adolescentes: estudo transversal, Passo Fundo, 2024. Epidemiol Serv Saude. 2026:35;e20250901.

**DOI:** 10.1590/S2237-96222026v35e20250901.a

**Published:** 2026-04-27

**Authors:** Keitty de Andrade

**Affiliations:** 1 Universidade de Brasília, Brasília, DF, Brazil Brasília DF Brazil

## Peer review A

## Questionnaire

Does the manuscript contain new and significant information to justify publication? Yes

Does the Abstract (Summary) clearly and accurately describe the content of the article? Yes

Is the problem significant and concisely stated? Yes

Are the methods described comprehensively? Yes

Are the interpretations and conclusions justified by the results? No

Is adequate reference made to other work in the field? Yes

## Manuscript Structure

Length of article is: Adequate

Number of tables is: Adequate

Number of figures is: Adequate

Please state any conflict(s) of interest that you have in relation to the review of this paper. None

Interest: Good

Quality: Average

Originality: Good

Overall: Average

Recommendation: Major Revision

## Comments

Prezados(as) autores(as),

O manuscrito é oportuno e aborda um tema de alta relevância. A seguir, apresento comentários detalhados, organizados por seção, com o objetivo de apoiar o aprimoramento do trabalho.

Título:

a) Recomenda-se revisar o título do manuscrito para torná-lo mais informativo e alinhado ao padrão adotado em estudos transversais. Títulos que seguem a estrutura “[Desfecho] em [população]: prevalência e fatores associados” facilitam a identificação do foco da pesquisa e sua aplicabilidade. Uma sugestão seria: “Sintomas de ansiedade e depressão em adolescentes: prevalência e fatores associados

Introdução:

• A introdução não deve se configurar como uma revisão da literatura sobre adolescência e saúde mental, mas sim apresentar de forma clara a justificativa para a realização da pesquisa. Sugere-se iniciar diretamente com o problema específico investigado, a elevada prevalência de sintomas de ansiedade e depressão em adolescentes e a necessidade de compreender seus fatores associados, como funcionalidade familiar e rede de apoio. Uma introdução mais concisa e focada no “por que” do estudo, destacando a lacuna de conhecimento e o impacto potencial para políticas públicas, tornará a leitura mais fluida e o propósito do trabalho mais evidente.

• Reformular o objetivo em uma frase única, clara, e idêntica ao resumo. No resumo está “Investigar prevalência de sintomas de ansiedade e depressão em adolescentes de 11 a 14 anos e associação com funcionalidade familiar e rede de apoio social.”

Métodos

A instrução é seguir o STROBE. Sugiro descrever o delineamento do estudo e inserir tópicos do STROBE no texto (ainda que provisoriamente no processo editorial, podendo remover após eventual aceite) e explicar cada procedimento dentro do tópico previsto, sem redundâncias e optando por linguagem direta. Veja abaixo a sequência que deve conter no método:

Desenho do estudo: Incluir de forma clara e destacada o delineamento do estudo logo no início da seção de métodos (ex: “Estudo transversal de base escolar, com coleta de dados primários…” etc.).

Contexto: Recomenda-se apresentar em parágrafo próprio o local do estudo (município, rede de ensino), a população-alvo, e o período completo de coleta de dados. Há uma inconsistência no texto quanto ao período de coleta: em um trecho é mencionado março a junho de 2024, e em outro, março a maio de 2024. Solicita-se uniformizar a informação ao longo do manuscrito e confirmar o período correto e realizar aqui a informação que está em “coleta de dados e instrumentos”.

Participantes: Os critérios de elegibilidade estão descritos, mas há necessidade de especificar como os estudantes foram selecionados: foi uma amostragem aleatória simples? por conglomerados? houve sorteio das escolas ou turmas?

Variáveis: Recomenda-se organizar e definir de forma mais clara e sistemática todas as variáveis do estudo: delimitar em parágrafo próprio que os desfechos principais são dicotômicos (presença/ausência de sintomas de ansiedade e de depressão, segundo pontos de corte definidos em escalas validadas); a funcionalidade familiar e a rede de apoio devem ser explicitamente identificadas como variáveis de exposição; as variáveis sociodemográficas (idade, sexo, escolaridade materna, autoavaliação de saúde) devem ser classificadas quanto ao seu papel na análise (preditoras, confundidoras ou apenas descritivas). Exemplo de formulação: “As variáveis X, Y e Z foram consideradas potenciais confundidores com base na literatura e foram incluídas nos modelos ajustados”.

Fontes de dados/mensuração: É necessário esclarecer se os instrumentos utilizados foram autoadministrados ou aplicados por entrevistador. O manuscrito menciona que os pesquisadores foram previamente treinados e estavam disponíveis para tirar dúvidas, o que sugere uma aplicação supervisionada, mas não está claro o grau de autonomia dos estudantes na resposta. Ainda, esclarecer se houve adaptação ou suporte específico para estudantes com condições especiais, como deficiências sensoriais, cognitivas ou transtornos de aprendizagem. Caso não tenha ocorrido tal demanda na amostra, essa informação também pode ser mencionada.

 Viés: O manuscrito não descreve quais medidas foram adotadas para minimizar possíveis fontes de viés. Recomenda-se explicitar: Que estratégias foram utilizadas para reduzir viés de seleção, como a aplicação padronizada em todas as escolas, taxa de resposta e critérios para exclusão de estudantes; Medidas para evitar viés de informação, considerando que o autorrelato de sintomas de saúde mental pode estar sujeito a sub ou supervalorização (ex: aplicação assistida, uso de instrumentos validados, ambiente reservado); Se houve algum cuidado com viés de desejabilidade social, comum em adolescentes ao responderem sobre saúde emocional e relações familiares.

 Tamanho do estudo: O cálculo amostral está parcialmente descrito, mas seria importante esclarecer: Qual desfecho principal foi utilizado no cálculo da amostra (ex: ansiedade ou depressão? Qual prevalência estimada?); Se o cálculo foi baseado em análise descritiva ou em modelo multivariado (importante dado o número de variáveis incluídas); Se foi considerado percentual de perdas ou recusas no cálculo final; Justificar a escolha do efeito de delineamento 1,20.

6. Métodos estatísticos: A regressão logística multinomial não é a abordagem mais apropriada para estimar razões de prevalência (RP). O uso de regressão de Poisson com variância robusta ou regressão binomial negativa seria mais indicado.

Resultados

Atender as diretrizes STROBE para esta seção também.

No texto da tabela 1, a expressão “a maior parte dos jovens informou ter mais de oito anos de estudo” está ambígua, pois quem tem escolaridade são as mães, e não os estudantes. Recomenda-se reescrever para indicar que se trata da escolaridade materna referida pelos estudantes.

No texto da figura 1, A expressão “relações distantes na família” pode ser mal interpretada. Na figura, os círculos concêntricos representam níveis de proximidade, mas isso não é explicitado no texto. Recomenda-se explicar que o centro representa relações muito próximas (como laços fortes de apoio), e os círculos externos representam relações mais periféricas, ainda que significativas. A expressão “relações distantes” poderia ser substituída por “relações periféricas” ou “menos próximas”.

No texto sobre a figura 2, há inconsistência entre as médias mencionadas no texto e os valores exibidos na figura. Por exemplo, o texto diz: “mais pessoas distantes na família (t = -3,162; p = 0,002)”. Porém, os dados da Figura 2a mostram 3,2 (sem sintomas) e 4,3 (com sintomas), então o valor mais alto é para quem tem sintomas, o que não combina com o sinal negativo do t. Além disso, expressões como “demonstrando menor possibilidade de suporte social” são interpretativas demais para uma seção de resultados. Sugere-se reformular para manter o tom descritivo apenas dos resultados e partes interpretativas podem ser realocadas para seção de discussão.

No texto sobre a figura 3, em alguns trechos, parece implicar relações de causa e efeito (ex.: “a funcionalidade familiar foi a variável que mais impactou…”). Reformular para expressar associação e não causalidade; Embora o texto mencione os sintomas de depressão, não fica claro se esse é o desfecho que a árvore está tentando prever ou se a árvore está apenas explorando agrupamentos; O texto afirma que “o modelo não identificou interações relevantes entre as variáveis ansiedade e depressão com o número de relações apoiadoras”, mas essa informação não é visível na figura.

O texto que descreve a Tabela 2 apresenta achados relevantes sobre os fatores associados aos sintomas de ansiedade e depressão entre adolescentes. No entanto, há inconsistências entre o conteúdo do texto e os dados efetivamente apresentados na tabela. Por exemplo, o texto menciona variáveis como escolaridade materna e autoavaliação de saúde, que não estão contempladas na Tabela 2. Caso essas variáveis tenham sido avaliadas na análise bruta, ainda que não tenham sido mantidas na análise ajustada, é recomendável incluí-las na tabela com os respectivos resultados da análise bruta. Caso contrário, sugere-se revisar o texto, suprimindo essas menções, a fim de evitar confusão; Além disso, há um erro conceitual na interpretação das variáveis relacionadas às relações apoiadoras. O texto afirma que “o número reduzido de relações apoiadoras no ambiente familiar foi fator de risco para sintomas de ansiedade e depressão (RP=1,06; IC95%: 1,01-1,34)”, mas esse valor de RP não aparece na tabela. Na verdade, a Tabela 2 mostra que a variável “relações familiares” foi significativamente associada aos desfechos, mas com uma razão de prevalência (RP) ajustada superior a 2, sugerindo que a ausência de apoio pode estar relacionada ao aumento da prevalência, não à proteção;

Optar por texto mais direto e evitar expressões como “Em relação à amostra dos estudos”

Traduzir para português palavras e expressões estrangeiras “node”. Quanto as palavras estrangeiras, a RESS adota a regra do Manual de Redação do Senado que dá uma lista de palavras que não recebem itálico. Fonte: https://www12.senado.leg.br/manualdecomunicacao/estilos/estrangeirismos-grafados-sem-italico-ou-aspas

Nomes próprios em língua estrangeira também não recebem itálico (nomes de instituições, universidades, aplicativos programas de computador, congresso etc.).

Observar orientações de redação e formato já solicitadas para seções anteriores e aplicar nos casos pertinentes desta seção.

Discussão

Seguir STROBE.

Recomenda-se que os autores resumam, logo no início da discussão, os dados centrais: a prevalência de sintomas de ansiedade e depressão, a associação com a funcionalidade familiar, as características das redes de apoio social e as diferenças observadas segundo o sexo.

Quanto às limitações do estudo, é importante que elas sejam destacadas de forma mais explícita e aprofundada. Atualmente, são mencionadas apenas no final da discussão, de forma sucinta. Sugere-se a elaboração de um parágrafo específico para essa finalidade, discutindo não apenas a limitação do delineamento transversal, que impede inferências causais, mas também outras possíveis fontes de viés, como o viés de informação decorrente da utilização de autorrelatos por adolescentes, o viés de seleção relacionado à inclusão exclusiva de estudantes matriculados em escolas, e possíveis limitações na mensuração das variáveis, sobretudo no que se refere à rede social significativa, cuja exclusão nos modelos estatísticos pode estar associada à forma como foi operacionalizada.

A interpretação dos resultados, embora bem articulada com a literatura, pode ser aprimorada ao considerar com maior cautela a magnitude e a direção das associações observadas. Seria importante que os autores comentassem, por exemplo, o grau de associação entre os níveis de disfuncionalidade familiar e os sintomas avaliados, considerando as medidas de efeito obtidas nas análises de regressão. Além disso, recomenda-se explicitar as limitações decorrentes da multiplicidade de análises realizadas, discutindo o risco de resultados espúrios. A comparação com outros estudos deve ir além da confirmação de achados semelhantes, incorporando também uma análise crítica das divergências, possíveis explicações para essas diferenças e implicações para a interpretação dos resultados.

Por fim, a discussão sobre a validade externa do estudo pode ser aprofundada. Os autores mencionam brevemente a limitação da amostra, restrita a um único município, mas seria interessante refletir sobre a possibilidade de generalização dos achados. Isso inclui discutir se a realidade sociodemográfica de Passo Fundo se assemelha a outros contextos urbanos brasileiros, se a faixa etária contemplada é representativa do espectro da adolescência, e em que medida os resultados podem ser utilizados para subsidiar políticas públicas em outras localidades.

Ilustrações

Remover ponto final de todos os títulos das ilustrações; ver regras de português com uso de maiúsculas, por exemplo “Funcionalidade Familiar”

Tabela 1:

Em algumas variáveis, como “funcionalidade familiar”, a ordem das categorias pode ser invertida para refletir uma escala de melhor para pior cenário, o que favorece a leitura intuitiva dos dados. Padronizar para as demais variáveis.

O título está compreensível, mas poderia ser ligeiramente ampliado para indicar que se trata de características sociodemográficas, de saúde e contexto familiar dos estudantes

Tabela 2:

sugere-se incluir na legenda da tabela uma descrição breve da análise realizada, como: “Modelos de regressão de Poisson com variância robusta foram utilizados para estimativa das razões de prevalência (RP), com ajuste para variáveis estatisticamente significantes.” Também é recomendável esclarecer o significado do asterisco () utilizado nos valores de RP, incluindo uma nota explicativa como: “ p < 0,05”.

Figura 1:

A legenda atual não explica o significado dos círculos concêntricos (níveis de proximidade) nem das cores usadas no texto (cores por sexo). Recomenda-se acrescentar uma legenda mais descritiva para que a figura seja autoexplicativa.

O título poderia ser ampliado para explicitar melhor o conteúdo.

Melhorar legibilidade da figura.

Figura 2:

Idem figura 1

Figura 3:

A legenda da figura deve ser mais informativa. É importante que o leitor compreenda o que representam os nós (nodes), os percentuais, as categorias de variáveis (ex.: “disfuncionalidade familiar severa” versus “moderada”), e o que é a variável-alvo da predição (sintomas de depressão?). Atualmente, o gráfico exige leitura do texto para interpretação, o que compromete a autonomia da figura.

Há inconsistência entre os termos usados no texto e na figura. Por exemplo, o texto menciona “funcionalidade familiar severamente disfuncional” e “alta funcionalidade”, mas na figura aparece “severamente disfuncional”e “altamente funcional”.

A figura parece ser resultado de uma árvore de decisão (ex.: CART ou CHAID), mas isso não está descrito na legenda nem indicado na figura.

As proporções apresentadas nos nós (percentuais) são relevantes, mas não está claro qual o denominador. São proporções de pessoas com depressão em cada grupo? Ou proporção do total da amostra?

Não está explicado na legenda nem no texto o que representam as letras “a”, “b”, “c”, “d”, “e” sob cada barra.

O que significam “com sintomas” e “sem sintomas” nas laterais dos gráficos de barras?

As expressões “com sintomas” e “sem sintomas” aparecem nas laterais de cada gráfico de barras, mas não está evidente se indicam o eixo Y, as categorias do eixo X ou uma legenda de cores. Além disso, o rótulo “Com sintomas de depressão” posicionado acima da barra, por exemplo no nó A, não é compatível com o conteúdo do gráfico, que mostra duas barras (uma preta e uma cinza). Isso causa confusão no leitor, já que, visualmente, a figura apresenta tanto adolescentes com sintomas quanto sem sintomas em cada barra, mas o título acima da barra menciona apenas um grupo.

Nomear eixo Y como “Proporção de adolescentes (%)”

Usar “nó” em vez de “node” para evitar anglicismos

Sobre o campo “Disponibilidade dos dados”: ajustar o texto e informar onde os dados de pesquisa podem ser acessados pelos leitores. Espera-se no item disponibilidade de dados, dados primários antes da análise dos dados ou síntese, exemplo um Excel com todos os manuscritos incluídos e todas as características, dados, e afins. Seria um banco de dados que foi gerado antes de fazer qualquer síntese de resultados. A Matriz ou qualquer figura não deve ser considerada um banco de dados e sim uma representação do dado, que deve ser ilustrada como figura/tabela principal ou material suplementar do manuscrito. Em um único Excel manter as informações de seleção, extração e qualidade metodológica e adicionar uma aba de dicionário de variáveis, caso alguma sigla ou redução de títulos tenha sido usada. Isso clarifica o entendimento do banco de dados. Então ficaria uma aba seleção, uma aba extração e qualidade e uma aba de dicionário de dados ou dois Excels um de seleção e outro de extração, mas ambos precisariam de uma aba de dicionário de dados, caso tenha siglas ou demais itens que precise de explicação. Ex de dicionário:

É necessário ainda que publique seus dados em uma plataforma como OSF, por exemplo. Lá é possível registrar um doi que será publicado no artigo. No texto escreva: Os conjuntos de dados gerados e analisados durante o estudo atual estão disponíveis no repositório de dados xxxx (doi com link).

Para orientar o aprimoramento do manuscrito, recomendo a consulta dos seguintes materiais, que apresentam orientações claras e objetivas sobre cada seção de artigos científicos:

Pereira, MG. Artigos científicos: como redigir, publicar e avaliar. Rio de Janeiro: Guanabara Koogan; 2011.

Pereira, MG. A introdução de um artigo científico. Epidemiol. Serv. Saúde v.21 n.4 Brasília out-dez. 2012. Disponível em: http://scielo.iec.pa.gov.br/scielo.php?script=sci_arttext&pid=S1679-49742012000400017&lng=pt&nrm=iso

Pereira, MG. A seção de método de um artigo científico. Epidemiol. Serv. Saúde v.22 n.1 Brasília jan-mar. 2013. Disponível em: http://scielo.iec.pa.gov.br/scielo.php?script=sci_arttext&pid=S1679-49742013000100020&lng=pt&nrm=iso

Pereira, MG. A seção de resultados de um artigo científico. Epidemiol. Serv. Saúde. v.22 n.2 Brasília abr-jun. 2013. Disponível em: http://scielo.iec.pa.gov.br/scielo.php?script=sci_arttext&pid=S1679-49742013000200017&lng=pt&nrm=iso

Pereira, MG. A seção de discussão de um artigo científico. Epidemiol. Serv. Saúde. v.22, n.3 jul-set. 2013. Disponível em: http://scielo.iec.pa.gov.br/scielo.php?script=sci_arttext&pid=S1679-49742013000300020&lng=pt&nrm=iso

Pereira, MG. O resumo de um artigo científico. Epidemiol. Serv. Saúde. v.22, n.4 out-dez 2013. Disponível em: http://scielo.iec.pa.gov.br/scielo.php?script=sci_arttext&pid=S1679-49742013000400017&lng=pt&nrm=iso&tlng=pt

